# Fear of hypoglycemia and illness perception in type II diabetes patients

**DOI:** 10.1186/s12902-024-01548-x

**Published:** 2024-02-20

**Authors:** Abdollahi Fariba, Mohammad Amerzadeh, Marjan Banazadeh, Saba Rashidi, Zahra Tayebi Myaneh

**Affiliations:** 1 Department of Medical Sciences, Qazvin Branch, Islamic Azad University, Qazvin, Iran; 2 Metabolic Diseases Research Center, Research Institute for Prevention of Non-Communicable Diseases, Qazvin University of Medical Sciences, Qazvin, Iran; 3 School of Nursing, Alborz University of Medical Sciences, Karaj, Iran; 4 Student Reserch committe, Alborz University of Medical Sciences, Karaj, Iran

**Keywords:** Type 2 diabetes, Fear of hypoglycemia, Illness perception, Predictors

## Abstract

**Background:**

Hypoglycemia, a prevalent acute complication among individuals with type 2 diabetes (T2D), manifests with varied symptoms. Those with diabetes who have previously encountered hypoglycemic episodes commonly develop a Fear of Hyperglycemia (FOH). Illness perception (IP) significantly affects self-care behaviors and health outcomes in individuals diagnosed with T2D.

**Objective:**

This study examined the correlation between IP and FOH among T2D patients and predictors of FOH.

**Methods:**

The present study employed a descriptive-analytical design. The target population for this investigation comprised patients diagnosed T2D who sought medical care at the clinic and endocrinology departments of a hospital affiliated with Alborz University of Medical Sciences. The data collection period spanned from August 2019 to March 2021. A total of 300 individuals were included in the sample. Questionnaires were administered to measure both IP and FOH. Statistical analysis was conducted to examine the association between IP and FOH, as well as to identify the predictors of FOH.

**Results:**

The results of the study indicated a statistically significant relationship between FOH and the mean score of IP among patients with diabetes (*p* = 0.001, *r* = 0.393), suggesting a moderate positive correlation between these variables. Additionally, the duration of illness, IP, and level of education were identified as variables that predicted FOH (*p* < 0.05).

**Conclusion:**

The numerous factors that influence FOH in individuals diagnosed with T2D highlight the necessity for strategic planning and training initiatives aimed at enhancing IP and reducing FOH within this specific population. Healthcare providers should prioritize interventions that not only address patients’ concerns but also contribute to the improvement of their overall well-being. By implementing such interventions, healthcare providers can optimize diabetes management strategies and ultimately enhance patient outcomes.

## Background

Globally, the prevalence of diabetes is a significant public health concern, affecting over half a billion individuals of all age groups, including men, women, and children. However, projections suggest that this number will more than double within the next three decades, reaching approximately 1.3 billion people, thereby impacting every country [1]. Furthermore, it is anticipated that the prevalence of diabetes will continue to rise, with estimates indicating a projected prevalence of 55% by the year 2035 [2]. Type 2 Diabetes (T2D), characterized as a chronic condition, is associated with both acute and long-term complications [3]. Among these complications, hypoglycemia is a prevalent acute occurrence in individuals with T2D, manifesting with various symptoms [4] such as dizziness, nausea, fatigue, sweating, shaking, pupil dilation, irritability, seizures, and coma. The prompt treatment of hypoglycemia is critical, as failure to do so can lead to severe consequences, including fatality [5]. Hypoglycemia is considered a medical emergency and is defined as a drop in blood glucose levels below 70 mg/dL [6]. Often, hypoglycemia can be attributed to an excessive dosage of oral antidiabetic drugs or insulin injections [7, 8].

Individuals with diabetes who have previously experienced hypoglycemic episodes often develop a sense of apprehension and fear regarding the potential recurrence of such events [9, 10]. This fear of hypoglycemia (FOH) has been found to have detrimental effects on illness management, metabolic control, and the ability of patients to maintain their regular activities, including work and social engagements, thereby impacting their overall quality of life (QOL) [11]. Moreover, FOH can negatively affect patients’ emotional well-being, leading to feelings of guilt, frustration, dependence on others, a perceived loss of control, and increased stress levels [12].

To mitigate the risk of hypoglycemia, patients can enhance their self-management skills, which encompass adherence to dietary and nutritional recommendations, weight and stress management, regular exercise, self-monitoring of glucose levels, and consistent medication adherence [13]. However, several factors can influence patients’ adherence to these self-care behaviors [14].

Empirical evidence strongly supports the idea that illness perception (IP) plays a critical role in influencing self-care behaviors and health outcomes in individuals with T2D [15]. Research studies have consistently demonstrated a significant association between diabetes perception and self-care practices, as well as QOL in individuals with T2D [16]. For instance, Broadbent et al. found a notable relationship between IP and adherence to medication, diet, and exercise among individuals with T2D. Their study also revealed that perceived personal control was a strong predictor of self-care behaviors in T2D patients [17]. Similarly, IP among T2D patients has been associated with body mass index, fasting blood glucose, total cholesterol, and blood pressure [18].

A previous review focusing on the correlation between IP and glycemic control, as measured by HbA1c levels, reported that more positive perceptions of control or cure were associated with better glycemic control [19]. Furthermore, a study conducted by Kugbey et al. found a significant positive correlation between IP and the level of psychological distress among individuals with T2D [15].

Indeed, research studies have identified various predictors of FOH among patients with T2D. Notably, FOH has been found to have a positive correlation with factors such as female gender, advanced age, and comorbid conditions including anxiety, stress, depression, and reduced QOL [20]. Additionally, the duration of diabetes has been identified as a predictor of FOH, indicating that longer disease duration may contribute to heightened fear [21]. Furthermore, a history of hypoglycemia has been associated with increased FOH [22]. Given that FOH serves as an indicator of glycemic control and is one of the primary drivers of T2D, it is crucial to investigate the factors associated with its development and persistence [23].

Examining the concept of IP in T2D, research has revealed a significant correlation between the perception of the disease and anxiety [24]. It appears that anxiety activates the autonomic nervous system [25], which can mask the symptoms of hypoglycemia. Consequently, individuals with anxiety may experience more frequent occurrences of hypoglycemic symptoms [26]. The repetition of such episodes can lead to a negative experience, further contributing to the development of FOH in these individuals. The association between anxiety and FoH has been demonstrated in several previous studies [27–30].

Based on the existing research and understanding, it is reasonable to hypothesize that anxiety acts as a mediator between IP and FOH [31]. Additionally, it is plausible to hypothesize that there will be a significant correlation between IP and FOH among patients with T2D.

## Objective

This study aimed to examine the correlation between IP and FOH among T2D patients as well as predictors of FOH.

## Methods

### Study design and participants

This is a descriptive- analytical study in endocrinology departments of a hospital affiliated with Alborz University of Medical Sciences between August 2019 and March 2021.

The inclusion criteria were individuals diagnosed with T2D for a year or longer, were between the ages of 30 and 65, were not pregnant women or individuals with a specific illness that would interfere with the study, were willing to participate, had no history of mental or psychological illness, and possessed minimum reading and writing skills. Participants who did not respond to more than 20% of the questions or were unwilling to continue were excluded from the study.

To estimate the sample size, the study referred to Momeni et al.‘s article [32]. According to that study, the mean FOH was reported as 16.8 with a standard deviation of 16.33. The desired type I error level was set at 0.05 (95% confidence level, α = 0.95-1). The error rate, denoted as d, was calculated as 0.113 multiplied by the standard deviation (d = 0.113*σ = 1.847). The sample size estimation was determined based on the following relationship

$$ n=\frac{{z}_{1-\frac{\alpha }{2}}^{2}*{\sigma }^{2}}{{d}^{2}}=\frac{{1.96}^{2}*{16.33}^{2}}{{1.847}^{2}}=\frac{1024.008}{3.413}=300$$

### Data collection

In this study, three questionnaires were used for data collection:

1. Demographic and clinical characteristics questionnaire: This questionnaire collected information on participants’ age, gender, level of education, marital status, occupation, type of drug used, duration of illness, complications of diabetes, and history of other diseases.
2. Brief Illness Perception Questionnaire (BIPQ): The BIPQ was used to assess various aspects related to T2D, including outcomes, duration, personal control, treatment control, nature, concerns, perception and recognition of the illness, emotional response, and perceived cause of the disease. The first eight questions were scored on a scale from 0 to 10, with reversed scoring for items 3, 4, and 7. The ninth question was an open-ended question asking the patient’s perspective on the three major causes of the disease. The total IP score was calculated by summing the scores of items 1 to 8, ranging from 0 to 80. Lower scores indicated a more positive IP. The scoring ranges for high, moderate, and low IPs were 0–27, 28–55, and 56–80, respectively. The Cronbach’s alpha coefficient for this questionnaire was 0.8, indicating good internal consistency, and the test-retest reliability coefficient ranged from 0.42 to 0.75 with a 6-week interval [33, 34].
3. Hypoglycemia Fear Survey (HFS) questionnaire: The HFS questionnaire was developed to assess FOH and related behaviors in diabetic patients [35]. The revised version used in this study, HFS-II, consists of 33 items and two subscales: Behavior (HFS-B) and Worry (HFS-W). The HFS-W includes 18 items that assess concerns related to hypoglycemia experienced in the past six months and its negative impacts. The items were rated on a 5-point Likert scale ranging from 0 (never) to 4 (always), with total scores ranging from 0 to 72. Higher scores indicate greater FOH. The validity and reliability of the HFS-II have been confirmed in previous studies, including one conducted in Iran [36–38, 32].

### Data analysis

The data were analyzed using statistical analysis and SPSS22 software. The normal distribution of the data was assessed using the Kolmogorov-Smirnov test. To investigate the relationship between FOH and IP and their related items, the Pearson correlation coefficient was utilized. Linear regression models were employed to explore the predictive factors of FOH. Specifically, the linear regression model examined how age, gender, level of education, diabetes treatment type, IP, and duration of illness predicted patients’ FOH. The significance level of the statistical tests was set at a *p*-value equal to or less than 0.05.

### Ethical considerations

This study obtained ethical approval from the Ethics Committee of Alborz University of Medical Sciences with the reference number IR.ABZUMS.REC.1398.172. To ensure compliance with ethical standards, the researcher obtained permission from the respective hospital and clinic authorities prior to commencing the study. Eligible patients were identified and provided with assurances regarding the confidentiality of their information. Detailed explanations were given to the participants on how to complete the questionnaires. Informed consent was obtained from each participant before collecting the necessary information. All ethical guidelines and protocols were followed throughout the study to protect the rights and well-being of the participants.

## Results

The statistical analysis included a total of 300 patients. Among the participants, the majority were female, accounting for approximately 54% of the sample. The majority of participants were also married, comprising around 90% of the total. Approximately 18.6% of patients reported having an academic education level or higher.

The mean age of the participants was 55.41 years with a standard deviation of 8.49 years. The duration of illness, measured in years, had a mean value of 9.57 years with a standard deviation of 5 years. Other clinical and demographic characteristics are presented in Table 1.

**Table 1:** Frequency distribution of demographic, social and illness characteristics of participants

Variables	Descriptive Statistics
**Gender n (%)**	
Male	138 (46)
Female	162 (54)
**Age Mean (SD)**	**55/41[8/49]**
**Marital status**	
Single	30 (10)
Married	270 (90)
Divorced	0(0)
**Occupation**	
Self-employment Housewife	110 (36.7)
Full time job	70 (23.3)
Unemployed	62 (20.7)
Retired	9 (3)
	70 (23.3)
**Level of education**	
Illiterate	56 (18.7)
Diploma- under diploma	188 (62.7)
Bachelor of Science	49 (16.3)
Master of Science-higher	7(2.3)
**Diabetes treatment type**	
**Insulin**	**30 (10)**
Antidiabetic oral medication	270 (90)
**Complications of diabetes**	
**No**	**14 (4.6)**
**Nephropathy**	**2 (0/66)**
**Retinopathy**	**24 (8)**
**Cardiovascular**	**9 (3)**
**Neuropathy**	**55 (18.3)**
More than 3 diseases	196 (65.3)
**History of other disease**	
**Yes**	**289 (96.3)**
No	11 (3.7)
**Duration of illness**	**9/57** (**5**)

The mean score of IP among the participants was 47.93 ± 6.69, indicating a moderate level of IP. The frequency distribution of IP scores revealed that 89.7% of the participants (*n* = 269) had moderate IP, while 10.3% (*n* = 31) had low IP.

A significant relationship was found between FOH and the mean IP score among patients with diabetes (*p* = 0.001, *r* = 0.393). Furthermore, the mean score of FOH among the participants was 22.02 ± 9.83, as presented in Table 2.

**Table 2:** Relationship between FOH and IP

Relationship of FOH	Impact of IP on life	IP duration	IP control	Perception of treatment impact	IP symptoms experience in life	Perception of concern about illness	Rate of illness recognition	IP impact on emotions	Total score of IP
*P*-value	0/001	0/823	0/085	0/001	0/001	0/001	0/866	0/001	0.001
R	0/284	0/013	0/104	-0/217	0/273	0/253	-0/009	0/323	0.393
Mean ± SD	8.25 ± 1.81	8.79 ± 1.77	2.53 ± 2.09	2.28 ± 1.85	7.82 ± 1.86	8.08 ± 1.81	1.95 ± 1.69	8.2 ± 1.72	47.93 ± 6.69

* Pearson test

The results showed a significant relationship between FOH and various subscales of IP, including the impact of IP on life, perception of treatment impact, IP symptoms experienced in life, perception of concern about illness, and IP impact on emotions (*p* < 0.001).

The linear regression analysis revealed that the duration of illness, IP, and level of education were significant predictors (*p* < 0.05) of FOH. Specifically, an increase in the duration of the disease was associated with higher levels of FOH. Patients with a bachelor’s degree exhibited less fear compared to those with a diploma or lower educational attainment, and patients with higher education demonstrated lower levels of fear compared to those with a diploma or lower education (*p* < 0.05). However, there were no statistically significant differences in fear levels between illiterate patients and those with a diploma or lower education (*p* > 0.05). [Table 3].

**Table 3:** Predictors FOH among patients with diabetes

		Non-standard regression coefficient	Standard error	Standard regression coefficient	*P*-value
IP		0.449	0.092	0.303	0.001
Age		0.085	0.085	0.066	0.35
Duration of diabetes [years]		0.376	0.14	0.193	0.007
Education	Illiterate	1.729	1.590	0.068	0.278
	Diploma- under diploma	1.780	1.570	0.05	0.265
	Bachelor of science	-3.768	1.874	-0.144	0.045
	Master of science-higher	-8.309	3.868	-0.0133	0.033
Diabetes treatment type	Insulin	-7.223	3.492	-0.186	0.004
	Oral drug	-1.293	1.358	-0.063	0.342

R^2^ = 0/289، R^2^adjusted = 0/222، F = 4/27، p- value < 0/05

## Discussion

This study examined the correlation between IP and FOH among T2D patients and predictors of FOH. In this study, participants’ IP was moderate. Our finding is similar with sina sabet et al. [2021] [14], but its different with nget et al. 2022. This difference in IP score can be due to differences in demographic factors, education, research location and other individual components [39].

The findings of this study suggest that the participants had lower levels of FOH. This finding is consistent with the results reported by Sakane et al. in 2019 and Hapunda and Pouwer in 2020, where the prevalence of FOH among participants was 27.7% and 19%, respectively [40, 41]. However, our study reported a slightly lower prevalence of FOH compared to these previous studies.

On the other hand, the prevalence of FOH reported in Majaliwa et al. in 2008 and Ahola et al. in 2016 was higher, with FOH levels of 55% and 52% among patients with diabetes, respectively [42, 43]. These differences in FOH prevalence may be attributed to variations in participant background characteristics and sample sizes across the studies. Factors such as cultural differences, healthcare systems, and individual experiences with diabetes management can also contribute to the discrepancies in FOH levels observed between studies.

### FOH and IP

The results of this study revealed a significant, positive, and direct relationship between IP and FOH in patients with T2D. Additionally, there was a significant positive correlation between FOH and several aspects of IP. These included the impact of IP on life, perception of treatment impact, IP symptoms experienced in life, perception of concern about illness, and IP impact on emotions.

No studies specifically investigating the correlation between IP and FOH in patients with T2D were found. However, there have been studies examining the relationship between IP and other variables. For instance, Sharry et al. (2012) conducted a study that explored the association between IP, glycemic control, and diabetes. Their findings indicated that perceiving greater serious effects of diabetes, attributing more symptoms to diabetes, being more concerned about diabetes, and having a greater emotional reaction to diabetes were significantly linked to poorer glycemic control. These results are consistent with our own findings, which demonstrated a positive association between these variables and FOH (indicated by higher HbA1c levels) [44]. This suggests that patients who perceive their disease as more threatening are more likely to experience FOH, which can lead to non-adherence to treatment and diet, ultimately resulting in elevated HbA1c levels.

Furthermore, other studies have identified a significant negative relationship between patients’ dietary practices, diabetes self-care practices, exercise, and IP [14]. Additionally, there is a positive relationship between IP and the level of psychological distress among T2D patients [15].

### Predictors FOH

The results of this study demonstrated that IP was a significant predictor of FOH in the population under investigation. Additionally, the duration of diabetes, education level, and insulin treatment were identified as predictors of FOH.

It is worth noting that several previous studies have also identified factors that contribute to the development of FOH. These factors include age, frequency of hypoglycemia, gender, and HbA1c levels [45–47]. However, in the present study, these specific variables did not predict the risk of FOH [45, 48, 49]. This discrepancy could be attributed to variations in study populations, methodologies, and other contextual factors.

### Strength and limitation of study

A strength of the present study was the relatively large sample size, which enhances the generalizability of the data. However, this study had several limitations, including its cross-sectional design, recruitment of participants from a single endocrinology and diabetes outpatient clinic, and the use of a self-administered questionnaire, which may be subject to response bias.

## Conclusion

In this study, diabetic patients had low levels of FOH. Correlated factor of FOH included IP. Therefore, providing infrastructure programs such as continuous education and appropriate care programs for diabetic patients to improve their IP can effectively improve their FOH.

## Data Availability

The datasets used and/or analyzed during the current study available from the corresponding author on reasonable request. The entire dataset is in Farsi language. The Data can be available in English language for the readers and make available from the corresponding author on reasonable request.
